# Rare Giant Granular Cell Ameloblastoma: A Case Report and an Immunohistochemical Study

**DOI:** 10.1155/2013/372781

**Published:** 2013-03-07

**Authors:** Santosh Hunasgi, Anila Koneru, Dinesh Singh Chauhan, Yadavalli Guruprasad

**Affiliations:** ^1^Department of Oral and Maxillofacial Pathology, Navodaya Dental College and Hospital, Raichur, Karnataka 584103, India; ^2^Department of Oral and Maxillofacial Surgery, AME's Dental College, Raichur, Karnataka 584103, India

## Abstract

*Aims*. The aim is to present a case of rare giant granular cell ameloblastoma and to review the pertinent literature highlighting the molecular aspects of its pathogenesis by analyzing the expression of CD-68, Bcl-2, and **β**-catenin. *Methods*. H and E stained sections showed large odontogenic islands showing peripheral ameloblast-like cells and central stellate reticulum-like cells with extensive granular cell transformation surrounded by fibrous stroma. Polyclonal rabbit anti-CD 68, anti-Bcl2, and anti-**β**-catenin were stained immunohistochemically. *Results*. CD-68 showed a moderate to strong staining intensity in granular cells. Moderate staining of Bcl-2 was expressed by the peripheral columnar cells of tumor islands and negative in the granular cells. Expression of **β**-catenin was generally weak, except for only the focal areas that showed a moderate staining intensity and weak in peripheral cells. *Conclusion*. The present case of giant granular cell ameloblastoma is a rare entity. Development of monstrous size is indicative of ameloblastomas persistent growth. Granular cell transformation in ameloblastomas probably occurs as a consequence of extensive molecular changes. Immunohistochemical studies help us to know the pathogenesis of this granular cell ameloblastoma. Therefore, an effort has been made here to study the expression of Bcl-2, CD-68, and **β**-catenin.

## 1. Introduction

 Ameloblastoma is the second most common odontogenic tumor, being clinically and histologically diverse. These tumors have several distinct clinical types, including solid, unicystic, desmoplastic, and peripheral ameloblastomas. Ameloblastomas are further subclassified as follicular, plexiform, granular, basal cell, and acanthomatous [1]. 

Granular cell ameloblastoma (GCA) is a less common histological subtype of ameloblastoma. The English language literature search has revealed approximately 30 studies regarding this rare subtype of ameloblastoma [2]. It is known to be locally aggressive among all the ameloblastomas and is important to separate GCA from other ameloblastomas because of higher incidence of malignancy and metastases [3]. 

The granular cells are rich in lysosomal granules, in which there is marked transformation of the cytoplasm, usually of stellate reticulum-like cells, so that it takes a very coarse, granular, and eosinophilic appearance. This “granular change” is thought to be due to a dysfunctional status of neoplastic cells, and the pathogenesis of this tumor seems to be age related. Thus, acquisition of granular cell phenotype has been attributed to an aging or degenerative change in long-standing lesions [4].

Ameloblastomas can show slow and asymptomatic growth, because of this, patient seeks treatment only after the lesion has grown remarkably large size [5]. Therefore neglected ameloblastomas may become enormous and cause gross facial deformities that pose considerable problems in management. In English language literature, 10 cases of giant ameloblastomas (3 cases of Follicular and 7 cases of plexiform) are reported till date [5, 6]. However, there are no cases of giant granular cell ameloblastomas reported. 

Thus the purpose of this paper is to present a case of Rare giant granular cell ameloblastoma and to review the pertinent literature highlighting the molecular aspects of its pathogenesis by analyzing the expression of CD-68, Bcl-2, and *β*-catenin in the present case. 

## 2. Case Report

A 39-year-old female patient reported to our hospital with a complaint of a large painless swelling over the left side of the face. She had first noticed the swelling 10 years ago which was painless with minimal extraoral manifestation. The patient neglected the swelling due to its painless nature and slow growth. 

**Figure 1:**
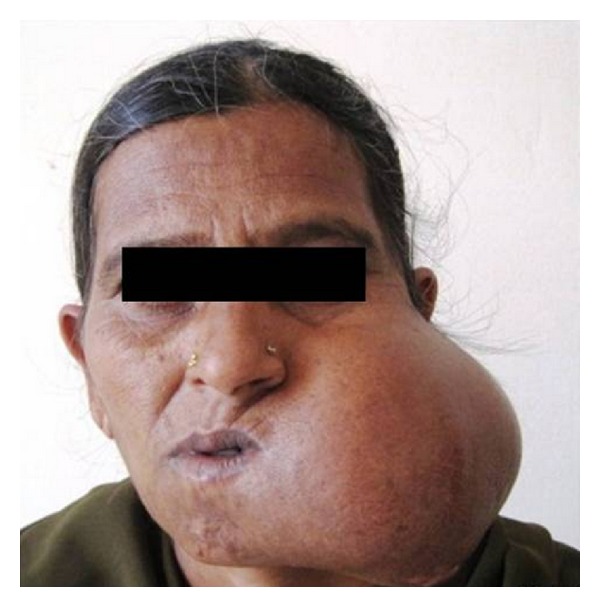
Extra oral examination showed a large well-defined swelling measuring approximately 12 × 9 × 10 cm in the left cheek, mandible and submental region.

On extra oral examination, a large well-defined swelling measuring approximately 12 cm × 10 cm was found in the left cheek, mandible, and submental region (Figure 1). Swelling was soft to firm in consistency with normal overlying stretched skin and no sinus or discharge observed. Intraorally, massive swelling was noticed from 31 to retromolar area, pushing the tongue to the contralateral side. Tongue movements were restricted. Crowding and extrusion of lower left anterior and premolars were observed.

**Figure 2:**
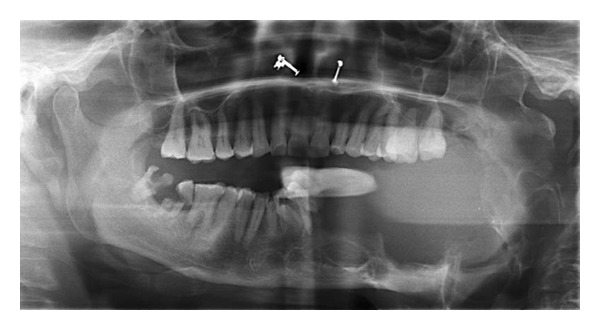
Radiographic examination revealed a multilocular radiolucency, extending from left condyle and coronoid process to right central and lateral incisors with thinning of lower border of mandible.

**Figure 3:**
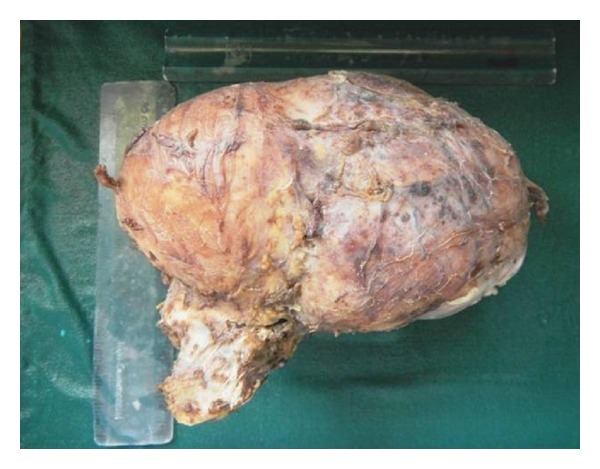
Surgically excised hemimandibulectomy specimen measured 12 × 9 × 10 cm.

Radiographic examination revealed a multilocular radiolucency, extending from left condyle and coronoid to right central and lateral incisors, with thinning of lower border of mandible (Figure 2). Surgically excised hemimandibulectomy specimen measured 12 × 9 × 10 cm and weighed 1200 grams (Figure 3). A followup of one year showed no recurrence. 

## 3. Methods

### 3.1. Histopathological Analysis

**Figure 4:**
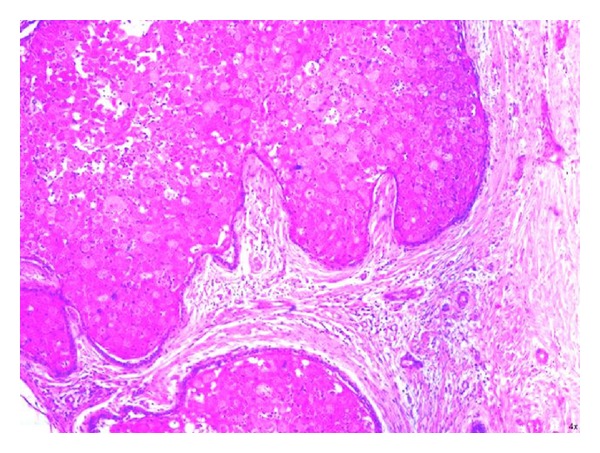
Large odontogenic islands showing peripheral ameloblast-like cells and central stellate reticulum-like cells with extensive granular cell transformation surrounded by fibrous stroma (H and E 20x).

**Figure 5:**
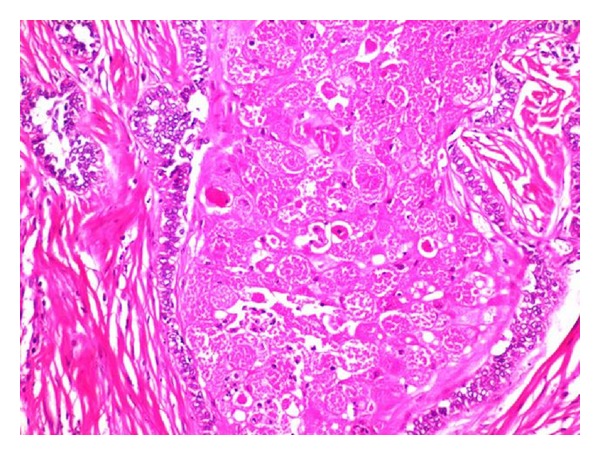
The granular cells exhibited coarsely granular eosinophilic cytoplasm and small pyknotic nuclei (H and E 40x).

H and E stained sections showed large odontogenic islands showing peripheral ameloblast-like cells and central stellate reticulum-like cells with extensive granular cell transformation surrounded by fibrous stroma (Figure 4). The granular cells exhibited coarsely granular eosinophilic cytoplasm and small pyknotic nuclei (Figure 5). Therefore, a final diagnosis of ameloblastoma, granular cell variant, was given. The patient was followed up for 1 year and showed no signs of recurrence.

### 3.2. Immunohistochemical Analysis

Polyclonal rabbit anti-CD-68, anti-Bcl-2, and anti-*β*-catenin (Biogenix Life Sciences Limited, CA, USA) were used for immunohistochemistry employing the super sensitive polymer HRP detection system. The sections were later counter-stained with Mayer's hematoxylin. The presence of brown color at the site of target antigen was considered as immunopositive. 

The immunoreactivity was assessed based on staining intensity. The staining intensity of positive cells was evaluated visually and classified as follows: weak, moderate, and strong intensity. Finally localization of positively stained cells was also assessed, that is, in peripheral ameloblast-like cells, central stellate reticulum like cells, and granular cells. 

## 4. Results

**Figure 6:**
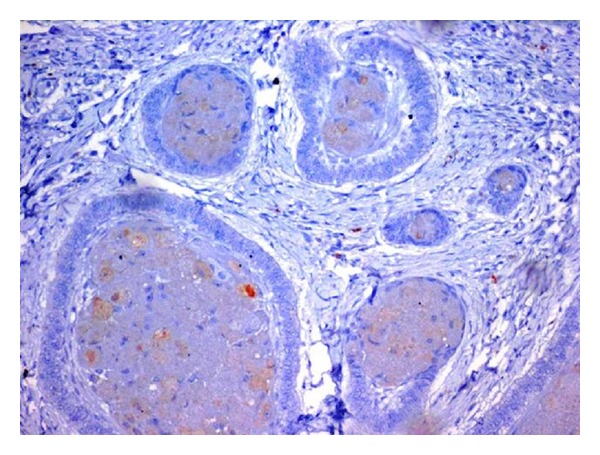
CD-68 showed a moderate to strong staining intensity in granular cells (20x).

**Figure 7:**
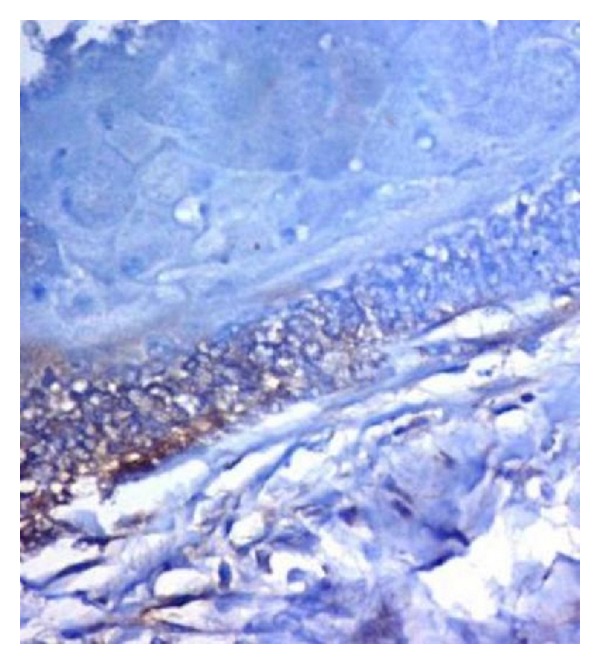
Moderate staining of Bcl-2 was expressed by the peripheral columnar cells of tumor islands and negative in the granular cells (40x).

**Figure 8:**
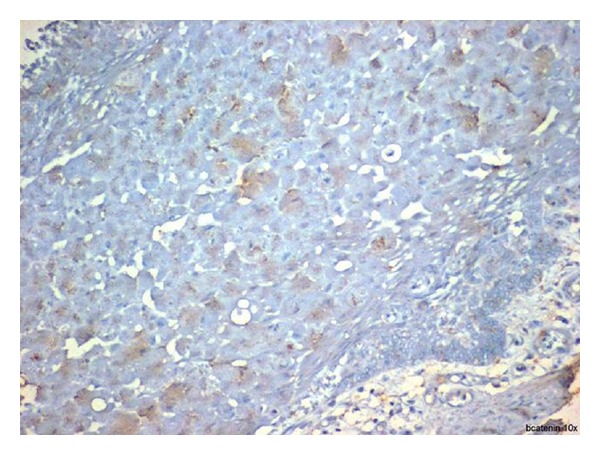
Expression of *β*-catenin was generally weak, except for only the focal areas that showed a moderate staining intensity and weak in peripheral cells (20x).

The tumor cells of granular cell ameloblastoma showed positive immunoreactivity to all proteins (Figures 6, 7, and 8). The expression of CD-68, Bcl-2, and *β*-Catenin is summarized in Table 1. CD-68 showed a moderate to strong staining intensity in granular cells (Figure 6). Moderate staining of Bcl-2 was expressed by the peripheral columnar cells of tumor islands and negative in the granular cells (Figure 7). Expression of *β*-catenin was generally weak, except for only the focal areas that showed a moderate staining intensity and weak in peripheral cells (Figure 8). 

## 5. Discussion

This study reports a case of granular cell ameloblastoma that developed at the posterior mandible of a 39-year-old female. Based on a series of 20 cases of granular cell ameloblastoma, this tumor occurs at an average age of 40.7 years with no significant gender predilection and is found predominantly at the posterior region of the mandible particularly around the angle of the mandible. The age and the location of tumor of this case appear to be consistent with the study by Hartman [7]. 

The term “giant” or “extreme” ameloblastoma is reserved for lesions that are truly large and that cause gross asymmetry and regional dysfunction. Patients with extreme ameloblastomas are usually from rural areas of developing countries who delay the treatment due to fear of surgery [5]. In the present case, the tumor reached an enormous size over 10 years causing gross facial deformity. Till date, there have been ten reported cases of extreme ameloblastoma (Table 2). The maximum size measured was 17 × 15 × 13 cm reported by Acharya et al. [5] where as present case measured 12 × 9 × 10 cm. All reports were of large tumors involving half of the mandible, and histological diagnosis in these ten cases was either follicular or plexiform type of ameloblastoma [5]. To our knowledge, this is the first reported case of giant granular cell ameloblastoma. 

Granular cell change in classic ameloblastoma is a well-recognized phenomenon. There has been considerable interest as to the nature of granular cells in ameloblastoma ever since it was recognized. The granular cells acquire small pyknotic nuclei and bulky cytoplasm filled with coarse eosinophilic granules indicating that there is an apoptotic process taking place. Several immunohistochemical studies showed increased apoptotic cells and decreased expression of antiapoptotic factors such as Bcl-2 and p53 proteins in granular cell ameloblastomas [8]. Similarly in the present case, Bcl-2 expression was negative in granular cells. 

In granular cell clusters, apoptotic cell fragments with condensed nuclei were phagocytosed by adjacent granular cells and degraded within lysosomes. Therefore, granular cells show positivity for CD-68, lysozyme, and alpha-1-antichymotrypsin indicating cytoplasmic lysosomal aggregates [8]. Similarly in the present case, CD-68 expression was moderate to strong in granular cells. 

Cell signaling pathways related to cell proliferation and differentiation are lost or inactive in the granular cells. Hence, the cell signaling molecules such as *β*-catenin, BMP-4, and Wnt-2 are altered in granular cells [9]. Similarly in the present case, *β*-catenin expression was shown to moderate staining in granular cells. Finally, the immunohistochemical results in the present case highlights that Bcl-2 negativity in granular cells indicating an apoptotic process, CD-68 positivity in granular cells indicating the presence of lysosomal aggregates and *β*-catenin cytoplasmic positivity in granular cells indicating an altered cell signaling pathways. 

Granular cell ameloblastoma is locally aggressive and has a relatively high chance of recurrence. Recurrence rate for granular cell ameloblastoma was reported to be 33.3%, which is higher, compared to the more common follicular, plexiform, and acanthomatous subtypes [10]. The present case was followed up for one year and showed no signs of recurrence.

## 6. Conclusion

The present case of giant granular cell ameloblastoma is a rare entity. Development of monstrous size is indicative of ameloblastomas persistent growth. Granular cell transformation in ameloblastomas probably occurs as a consequence of extensive molecular changes occurring in central stellate reticulum-like cells. Immunohistochemical studies help us to know the pathogenesis of this granular cell ameloblastoma. Therefore, an effort has been made here to study the expression of Bcl-2, CD-68, and *β*-catenin. Further studies on a large scale are essential for better understanding of the molecular pathogenesis of ameloblastoma and its subtypes in providing diagnostic and therapeutic benefits.

## Figures and Tables

**Table 1:** The expression of CD 68, Bcl2, and *β*-catenin in the cytoplasm of tumor cells in a granular cell ameloblastoma.

Examined proteins	Staining intensity		
	Peripheral cells	Central cells	Granular cells
CD 68	No staining	Weak	Moderate to severe
Bcl2	Moderate to severe	No staining	No staining
*β*-Catenin	Weak	Moderate to severe	Moderate

**Table 2:** A review of clinical features, tumor size, and histopathological type of giant ameloblastomas that were previously reported and the present case.

SL. no	Year	Age	Sex	Tumor size	Histopathological type
1	1977	57	M	Mental region to ramus of mandible	Follicular ameloblastoma
2	1977	62	F	Right wisdom tooth to left 1st premolar tooth region	Plexiform ameloblastoma
3	1985	30	F	15 × 14 × 12 cm	Plexiform ameloblastoma
4	1990	33	M	Right cuspid tooth to left mandibular condyle	Follicular ameloblastoma
5	1991	39	F	11 × 10 × 6 cm	Plexiform ameloblastoma
6	1995	73	M	11 × 11 × 14 cm	Plexiform ameloblastoma
7	1997	62	F	17 × 15 × 13 cm	Plexiform ameloblastoma
8	1999	53	F	15.2 × 11.4 × 12 cm	Plexiform ameloblastoma
9	2005	53	M	14 × 11 × 10 cm	Follicular ameloblastoma
10	2011	35	F	15 × 12 × 10 cm	Plexiform ameloblastoma
11	Present case	39	F	12 × 9 × 10 cm	Granular cell ameloblastoma
